# No nosocomial transmission under standard hygiene precautions in short term contact patients in case of an unexpected ESBL or Q&A *E. coli* positive patient: a one-year prospective cohort study within three regional hospitals

**DOI:** 10.1186/s13756-017-0228-6

**Published:** 2017-06-26

**Authors:** Dennis Souverein, Sjoerd M. Euser, Bjorn L. Herpers, Corry Hattink, Patricia Houtman, Amerens Popma, Jan Kluytmans, John W. A. Rossen, Jeroen W. Den Boer

**Affiliations:** 1 Department of Epidemiology and Infection Prevention, Regional Public Health Laboratory Kennemerland, Boerhaavelaan 26, 2035 RC, Haarlem, The Netherlands; 2 Department of Infection Prevention, Rode Kruis Ziekenhuis, Beverwijk, The Netherlands; 3 Department of Infection Prevention, Spaarne Gasthuis, Haarlem and Hoofddorp, The Netherlands; 4 Laboratory for Microbiology and Infection Control, Amphia Hospital, Breda, The Netherlands; 5 University Medical Center, Utrecht, The Netherlands; 6 Department of Medical Microbiology, University of Groningen, University Medical Center Groningen, Groningen, The Netherlands

**Keywords:** HR-GNRs, ESBL, Transmission, Contact tracing, Contact isolation

## Abstract

**Background:**

Many Highly Resistant Gram Negative Rod (HR-GNR) positive patients are found unexpectedly in clinical cultures, besides patients who are screened and isolated based on risk factors. As unexpected HR-GNR positive patients are isolated after detection, transmission to contact patients possibly occurred. The added value of routine contact tracing in such situations within hospitals with standard hygiene precautions is unknown.

**Methods:**

In 2014, this study was performed as a prospective cohort study. Index patients were defined as those tested unexpectedly HR-GNR positive in clinical cultures to diagnose a possible infection and were nursed under standard hygiene precautions before tested positive. After detection they were nursed in contact isolation. Contact patients were still hospitalized and shared the same room with the index patient for at least 12 h. HR-GNR screening was performed by culturing a rectal and throat swab. Clonal relatedness of HR-GNR isolates was determined using whole genome sequencing (WGS).

**Results:**

Out of 152 unexpected HR-GNR positive patients, 35 patients (23.0%) met our inclusion criteria for index patient. ESBL *E. coli* was found most frequently (*n* = 20, 57.1%), followed by Q&A *E. coli* (*n* = 10, 28.6%), ESBL *K. pneumoniae* (*n* = 3, 8.5%), ESBL *R. ornithinolytica* (*n* = 1, 2.9%) and multi resistant *P. aeruginosa* (*n* = 1, 2.9%). After contact tracing, 69 patients were identified as contact patient of an index patient, with a median time between start of contact and sampling of 3 days. None were found HR-GNR positive by nosocomial transmission.

**Conclusions:**

In a local setting within hospitals with standard hygiene precautions, routine contact tracing among unexpected HR-GNR positive patients may be replaced by appropriate surveillance as we found no nosocomial transmission in short term contacts.

## Background

Infections with Highly Resistant Gram Negative Rods (HR-GNRs) are associated with higher (hospital) costs, morbidity and mortality in comparison to susceptible micro-organisms [1–4]. Increasingly, studies report on the (colonization) prevalence of HR-GNRs, including ESBL (Extended Spectrum Beta Lactamase) producing bacteria isolated from hospitalized patients, general practitioner patients and nursing home residents [5–9]. Knowledge about regional prevalence rates is important since HR-GNR colonized patients constitute a potential reservoir for patients at risk for nosocomial infections, such as immune compromised patients and/or patients with open wounds [10–13]. Several studies showed that foreign travel is an important risk factor for HR-GNR colonization [14–16]. Therefore, in Dutch hospitals, patients who have a recent history of foreign hospital admission are actively screened and pre-emptively isolated until test results are known [17]. In addition, known HR-GNR positive patients are flagged in the Hospital Information System (HIS) and isolated when readmitted. Despite screening and isolation of high risk patients, numerous patients are found unexpectedly HR-GNR positive in clinical cultures to diagnose a possible infection [18]. Before detection these unexpected positive patients were not nursed in isolation so that transmission to other patients may have occurred since no specific infection control measures were taken except standard hygiene procedures.

Willemsen et al. showed that the nosocomial transmission rate of HR-GNRs in Dutch hospitals was 7.0%, using AFLP (Amplification Fragment Length Polymorphism) to determine the genetic relation between clinical isolates [18]. Tschudin-Sutter et al. showed that nosocomial transmission from unexpected ESBL positive patients to contact patients rarely occurs, with a transmission rate of 2.2% over a total study period of 11 years [19]. Based on these studies it could be questioned if contact tracing within hospitals with standard hygiene precautions is required as contact tracing is considered time consuming and expensive. For the development of future health policies the results of such studies are of major importance.

In the present study, the nosocomial transmission rate from unexpected HR-GNR positive patients to contact patients was studied within three regional hospitals in the Dutch region Kennemerland. In addition, we estimated the overall HR-GNR incidence including patients who were screened and pre-emptively isolated at admission.

## Methods

### Study design and setting

The present study was performed as a prospective cohort study. Three hospitals in the region Kennemerland participated in this study. Hospital one is a 260-bed regional hospital (37% private, 26% double and 37% multi-patient rooms), hospital two is a 400 bed teaching hospital (50% private, 25% double and 25% multi-patient rooms) and hospital three is a 400 bed teaching hospital (46% private, 37% double and 17% multi-patient rooms). A database was created including patient and laboratory information from index and contact patients. Data were collected in 2014 as part of each hospitals infection control program.

### Definition of HR-GNR

HR-GNR definitions were based on the Dutch MDRO (Multi-Drug Resistant Organism) directive for hospitals [18, 20]. HR-GNRs considered in the present study were (1) Enterobacteriaceae that were Extended Spectrum Beta-Lactamase (ESBL) and/or carbapenemase positive (CPE) and/or resistant to Fluoroquinolones and Aminoglycosides (Q&A), (2) *Acinetobacter* species that were carbapenemase positive and/or resistant to Q&A, (3) *Stenotrophomonas maltophilia* resistant to co-trimoxazole and (4) multi-resistant *Pseudomonas aeruginosa*, defined as resistant to at least three of the following antibiotics or antibiotic groups: piperacillin, ceftazidime, fluoroquinolones, aminoglycosides and/or carbapenemase positive.

### Definition of index patients, contact patients and infection control procedures

Independent of the sampled body site, patients who tested unexpectedly HR-GNR positive in clinical cultures were considered as index patient. Unexpected positive was defined as patients who were not earlier identified as HR-GNR carrier (not flagged in the HIS) and/or not screened because of an elevated risk at admission (history of foreign hospital admission or coming from a hospital with a known HR-GNR problem). Index patients were identified by the infection control department based on daily communicated laboratory results. Contact patients were defined as patients who were still hospitalized and shared the same room with the index patient for at least 12 h, while the index patient was nursed under standard hygiene precautions, which includes wearing gloves after entering the patients room (before performing any patient-care activity) and wearing an apron when handling contagious materials. Hand hygiene was performed according to the five moments of the WHO hand hygiene guideline [21]. Screening of contact patients was performed by sampling a rectal and throat swab (Copan eSwab including 1 mL of modified liquid Amies) supplemented with wound samples when present as soon as possible after detection of the index patient. Index patients with at least one contact patient (still hospitalized at the time of detection) were included in the study. After detection, all HR-GNR positive patients were nursed in contact isolation following the national MDRO directive [18]. Contact isolation consisted of nursing in a single room, using gloves by nursing personnel and daily disinfection of the patient room. For all HR-GNR positive patients, isolation measures were maintained during the total admission time and study period. HR-GNR positive patients were not unmarked during the study period and an alert was entered in the HIS as a warning when patients were readmitted.

### Sampling of patients and laboratory techniques

All samples were processed and analysed using Standard Operating Procedures (SOPs) at the Regional Public Health Laboratory Kennemerland (RPHLK). Samples from unexpected HR-GNR positive patients (index patients) were analysed using standard microbiological procedures. When the index patient was positive for a HR-GNR (including ESBLs) rectal and throat swabs from contact patient(s) were analysed by direct culturing on both an ESBL screening agar (ChromID ESBL-ID, bioMerieux, enriched with a mixture of antibiotics, including cefpodoxime) and a CLED GM20 agar (cystine lactose electrolyte deficient agar with 20 mg/L gentamicin, Oxoid). All gram-negative rods growing on these agars were identified using MALDI-TOF (Bruker Daltonics, Germany). Antibiotic susceptibility testing was performed using the automated system VITEK2 (bioMérieux, France). All isolates suspected for the production of ESBL, defined as a VITEK 2 AES alert and/or elevated MIC (> 1 mg/L) for cefotaxime and/or ceftazidime were confirmed using the combination disk method (ceftazidime and cefotaxime or cefepime with and without clavulanic acid). Isolates with a VITEK 2 AES alert and/or elevated MIC for meropenem (> 0.25 mg/L) were suspected for carbapenemase production. Carbapenemase production was analysed using the modified Hodge test and an in-house carbapenemase PCR with targets for KPC, VIM, OXA-48 and NDM [22–24]. All HR-GNR positive isolates were stored at −80 °C.

### Molecular typing of HR-GNR positive isolates

Isolates with similar micro-organism and HR-GNR type within index and contact patient(s) were genotyped using Whole Genome Sequencing (WGS) using the MiSeq instrument (Illumina) as described elsewhere [25]. De novo assembly was performed using CLC Genomics Workbench v7.0.3 (CLC bio A/S, Aarhus, Denmark) after quality trimming (Qs ≥ 28) with optimal word sizes based on the maximum N50 value. The sequence type (ST) was identified by uploading the assembled genomes to the multilocus sequence type (MLST) server (version 1.7) and the acquired resistance genes were determined with the CGE Resfinder 1.2 tool. Pairwise genetic distance between isolates was calculated for whole genome (wgMLST) targets and core genome (cgMLST) targets by dividing the number of allele differences by the total number of targets shared by both sequences and reported as proportion. Based on previously described methods, *E. coli* isolates with a genetic distance of 0.95% or less were interpreted as clonally related [25].

### Data analysis and definition of transmission

The transmission rate from index patients to contact patients was calculated by dividing the number of confirmed positive contact patients by the number of index patients, including those for whom no transmission had occurred. For every index and contact patient the following variables were calculated: contact time, defined as the period that the index patient and contact patient shared the same room; admission time; and time to sampling for the contact patient (after identification of the index patient). The overall cumulative HR-GNR incidence and incidence density for the study period was calculated by dividing the number of HR-GNR positive hospitalized patients by the total number of admissions and (hospital) patient-days. Confidence intervals (95%) for proportions were calculated using the Wilson score [26]. All statistical analyses were performed using IBM SPSS Statistics version 24.0.

## Results

### Characteristics of index and contact patients

**Fig. 1:**
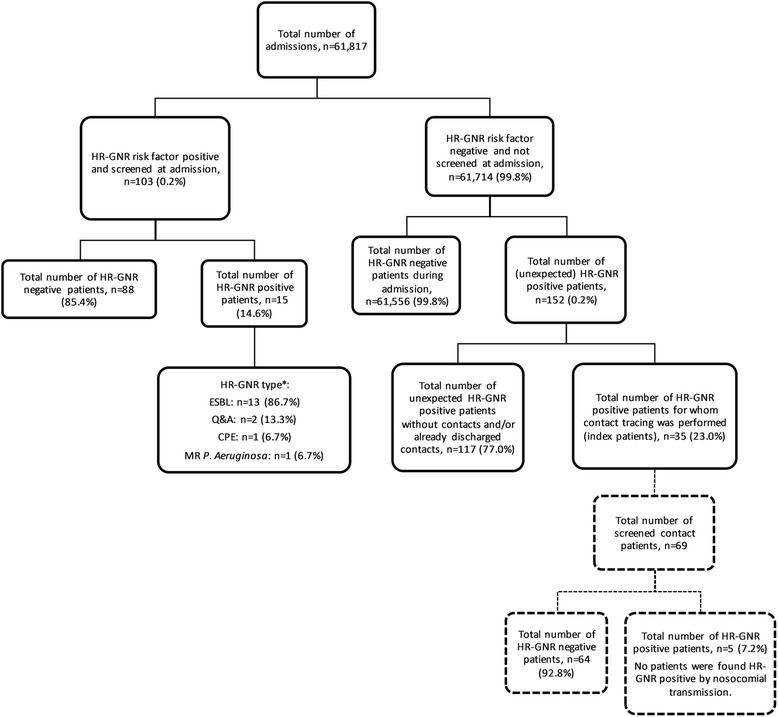
Flowchart of admitted patients, HR-GNR screening and unexpected HR-GNR positive patients. *Two patients were positive for two HR-GNR types (one patient was ESBL and CPE positive and one patient was ESBL and multi-resistant *P. Aeruginosa* positive)

Thirty-five out of 152 unexpected HR-GNR positive patients (23.0%) met our inclusion criteria and were marked as index patient. Consequently, 117 HR-GNR positive patients (77.0%) were excluded since no contact patients were identified or were already discharged. Around these index patients 69 patients were identified as contact patient (Fig. 1). Two contact patients (2.9%) were screened since they had contact on the Intensive Care Unit (ICU) with an index patient. The median number (range) of contact patients per index patient was 2 (1–5) and the median contact time (range) between index and contact patients was 2 days (0.5–9). Thirteen out of the 35 index patients (37.1%) and 44 out of the 69 contact patients (63.8%) were male and the mean age (SD) for index and contact patients was 72.1 (12.0) and 70.7 (15.2) years, respectively. The median admission time (range) for index and contact patients was 10 (2–36) and 11 (1–133) days and the median number of (hospital room) transfers (range) for index and contact patients were 3 (1–8) and 2 (1–9) transfers, respectively. The number of contact patients who used antibiotics and/or had open wounds at the time of sampling was 25 (36.2%) and 13 (18.8%) respectively. Stratified patient characteristics per hospital are shown in Table 1.

**Table 1:** Characteristics of index and contact patients

	Regional		Hospital 1		Hospital 2		Hospital 3	
	Index	Contacts	Index	Contacts	Index	Contacts	Index	Contacts
Number of patients	35	69	13	35	8	13	14	21
Sex								
Male (%)	13 (37.1%)	44 (63.8%)	5 (38.5%)	21 (60.0%)	4 (50.0%)	7 (53.8%)	4 (28.6%)	16 (76.2%)
Mean age (SD)	72.1 (12.0)	70.7 (15.2)	73.3 (11.5)	74.9 (14.7)	65.9 (13.7)	70.3 (13.3)	74.5 (11.0)	64.0 (15.1)
Median number of contacts (range)	2 (1–5)	NA	3 (1–4)	NA	1 (1–5)	NA	1 (1–3)	NA
Median admission time in days (range)	10 (2–36)	11 (1–133)	10 (2–24)	10 (2–48)	11.5 (5–32)	12 (8–29)	8 (4–36)	12 (1–133)
Median number of transfers (range)	3 (1–8)	2 (1–9)	3 (2–8)	2 (1–9)	3.5 (2–8)	3 (2–6)	2 (1–4)	2 (1–5)
Median contact time in days (range)	NA	2 (0.5–9)	NA	1 (0.5–6)	NA	4 (1–6)	NA	3 (0.5–9)
Median time between end of contact and sampling (range)	NA	1 (−2–12)	NA	1 (−1–8)	NA	2 (0–12)	NA	0 (−2–7)
Wounds (%)	NA	13 (18.8%)	NA	6 (17.1%)	NA	2 (15.4%)	NA	5 (23.8%)
Antibiotic use during sampling (%)	NA	25 (36.2%)	NA	13 (37.1%)	NA	6 (46.2%)	NA	6 (28.6%)

*NA* not applicable

### HR-GNR types and micro-organisms of ‘index patients’

Of all 35 index patients, 20 patients were found ESBL *E. coli* positive (57.1%), followed by Q&A *E. coli* (*n* = 10, 28.6%), ESBL *K. pneumoniae* (*n* = 3, 8.5%), ESBL *R. ornithinolytica* (*n* = 1, 2.9%) and multi resistant *P. aeruginosa* (*n* = 1, 2.9%).

### Transmission analysis

In total, five out of 69 contact patients (7.2%) were found HR-GNR positive. All of these contact patients were associated with a different index patient. Four of these patients were ESBL *E. coli* positive and one patient was positive for a Q&A *E. coli*. Three of the five HR-GNR positive contact patients were positive with a different HR-GNR type and/or micro-organism compared to their index patient and were therefore considered negative for nosocomial transmission. Two of the five HR-GNR positive contact patients were positive with the same HR-GNR type and micro-organism as their index patient (both ESBL *E. coli*). WGS of the ESBL *E. coli* isolates of index 1 and contact 1 showed that the isolate of index 1 was genotyped as ST 131, CTX-M-27. The isolate of contact 1 was genotyped as ST 295, SHV-12. The genetic difference between these isolates was determined using cgMLST and wgMLST and showed a genetic difference of 95.8% and 97.5% (Table 2). WGS of the ESBL *E. coli* isolates of index 2 and contact 2 showed that the isolate of index 2 was genotyped as ST 69, CTX-M-1 and the isolate of contact 2 as ST 69, CTX-M-55. The genetic difference using cgMLST was 11.6%, and 13.6% when using wgMLST (Table 2). Based on the WGS data we concluded that nosocomial transmission had not occurred in both cases. Consequently, the overall nosocomial transmission rate (95% CI) of unexpected HR-GNR and HR-GNR *E. coli* positive patients to contact patients was 0% (0–9.9) and 0% (0–11.4).

**Table 2:** Index patients with possible transmission

Pair	Index	Contact	Genetic difference (wgMLST)	Genetic difference (cgMLST)	Conclusion
1	*E. coli* (ST131; CTX-M-27)	*E. coli* (ST295; SHV-12)	97.5% (3138/3219)	95.8% (2647/2764)	No nosocomial transmission
2	*E. coli* (ST69; CTX-M-1)	*E. coli* (ST69; CTX-M-55)	13.6% (439/3219)	11.6% (321/2764)	No nosocomial transmission

*ST* sequence type, *wgMLST* whole genome multilocus sequence typing, *cgMLST* core genome multilocus sequence typing

### Incidence of HR-GNRs

In 2014, 15 out of 103 patients (14.6%) were HR-GNR positive as part risk factor based screening at admission (Fig. 1). In addition, 152 patients were unexpected HR-GNR positive in clinical cultures during hospitalization. Together with five positive contacts this resulted in a total of 172 HR-GNR positive patients during hospitalization. Given a total of 61,817 admissions and 223,351 (hospital) patient-days this resulted in a cumulative incidence (95% CI) and incidence density (95% CI) of 27.8 (24.0–32.3) patients per 10,000 admissions and 7.7 (6.6–8.9) patients per 10,000 (hospital) patient-days, respectively. As shown in Table 3, 68.6% (*n* = 118) of all HR-GNR positive patients tested positive for an ESBL. We expect based on the detected prevalence within contact patients (7.2%) that 4450 admissions were with patients that were HR-GNR colonized (7.2% of 61,817 admissions).

**Table 3:** Total HR-GNR incidence for 2014

	Regional	Hospital 1	Hospital 2	Hospital 3
ESBL	118	40	37	41
Q&A	54	14	20	20
CPE	3	1	0	2
*S. maltophilia* resistant to co-trimoxazole	1	1	0	0
MR *P. aeruginosa*	5	3	1	1
Total number of HR-GNR	181	59	58	64
Number unique HR-GNR positive patients^a^	172	53	57	62
Number of admissions	61,817	18,837	23,637	19,343
Number of patient days	223,351	57,749	92,070	73,533
HR-GNR incidence rate per 10.000 admissions (95% CI)	27.8 (24.0–32.3)	28.1 (21.5–36.8)	24.1 (18.6–31.2)	32.1 (25.0–41.1)
HR-GNR incidence density per 10.000 patient-days (95% CI)	7.7 (6.6–8.9)	9.2 (7.0–12.0)	6.2 (4.8–8.0)	8.4 (6.6–10.8)

^a^Represents the number of unique patients. During admission, a patient could be positive for more than one HR-GNR type. Therefore, this number is lower than the sum of all HR-GNR subgroups

*HR-GNR* Highly Resistant Gram Negative Rod, *MR* multi resistant, *ESBL* extended spectrum beta lactamase, *Q&A* enterobacteriaceae or *Acinetobacter* spp. resistant to fluoroquinolones and aminoglycosides, *CPE* carbapenemase producing enterobacteriaceae

## Discussion

During a study period of 1 year, a nosocomial transmission rate of 0% from unexpected HR-GNR positive patients to contact patients was found. Out of 152 unexpected HR-GNR positive patients, 35 patients met our inclusion criteria for index patients. Around these 35 index patients, 69 contact patients were sampled, accounting for a total of 178 contact days. Although no nosocomial transmission had occurred, five contact patients were HR-GNR positive (7.2%) and four of these were ESBL *E. coli* positive (5.8%), which corresponds with earlier reported prevalence rates in Dutch hospitals [6, 27]. As expected, ESBL positive patients were found most frequently among all HR-GNR positive patients (68.6%) and index patients (68.6%). From a micro-organism perspective, 85.7% of the index patients were positive for an HR-GNR *E. coli*. MRSA, VRE (Vancomycin-resistant *Enterococcus*) and PRSP (Penicillin-resistant *Streptococcus pneumoniae*) were not included in the present study.

Other studies with comparable study designs that estimated the transmission rate to contact patients are scarce, limiting the comparison with other settings. Willemsen et al. showed that the nosocomial transmission rate of HR-GNR in Dutch hospitals was 7.0% [18]. This is probably a worst-case scenario since only epidemiologically linked clinical isolates within a time window of 4 weeks were analysed using AFLP genotyping, which is considered less discriminatory. In 2012, Tschudin-Sutter et al. studied the transmission rate from unexpected ESBL positive patients to contact patients [19]. Their results showed that during a period of 11 years two contact patients related to 93 index patients (2.2%) were ESBL positive by transmission, suggesting that nosocomial transmission rarely occurs. A study performed at the ICU in a French hospital showed an ESBL acquisition rate of 6.5% [28]. However, only one patient (out of 19) appeared to be positive by nosocomial transmission. A complicating factor for these studies (and also for our study) is the relatively high ESBL colonization prevalence in the community. We only detected 172 of these patients during our study period instead of an expected amount of 4450 admissions with HR-GNR positive patients. Consequently, expensive high resolution genotyping is needed to exclude transmission since phenotypic results, MLST, AFLP or ESBL gene are not able to discriminate enough between closely related isolates [25, 29]. Based on MLST and ESBL group alone we would have concluded that transmission had occurred between one index and a contact patient. Additional cgMLST or wgMLST analyses, as performed in the present study minimizes the chance on this kind of false conclusions. Another interesting study within a German hospital showed a nosocomial transmission rate of 2.3% for multidrug resistant *E. coli* based on clinical (infection) isolates using cgMLST [30]. When isolation measures of positive patients were ceased the transmission rate increased non-significantly to 5.0% and decreased on high risk wards (ICU). However, as these results were based on clinical infection cultures only, colonized (not infected) patients were missed, underestimating the real transmission rate.

Our results and the previously mentioned studies clues that routine contact tracing in case of an unexpected HR-GNR positive patient might be replaced by appropriate surveillance in a local setting within hospitals with standard hygiene precautions. Also, since we found no nosocomial transmission, these results advocate a more flexible isolation strategy. However, (cluster) randomized controlled trials are needed to compare nosocomial transmission rates between different isolation strategies. Preferably such studies must be accompanied by adverse events that are associated with isolation (such as patient well-being) so that a balanced conclusion could be made. Because we mainly isolated HR-GNR *E. coli*, our results should be interpreted with caution and cannot simply be generalized for less frequently isolated HR-GNRs such as CPE or other micro-organisms than *E. coli* such as *K. pneumoniae*.

Some studies have suggested that certain sequence types of *E. coli* (ST 131) and *K. pneumoniae* (ST 258) are hyperendemic, causing outbreaks and infections [31, 32]. A recent review found evidence that *E. coli* ST 131 is more pathogenic than non-ST131, but the increased transmissibility or prolonged carriage could not be confirmed [33]. For *K. pneumoniae* ST 258, this study could not confirm or reject the increased pathogenicity, transmissibility or prolonged carriage of this sequence type [33]. As certain HR-GNR types or micro-organisms are potentially more dangerous in terms of transmissibility or pathogenicity, contact tracing can only be replaced in a local setting within hospitals where adequate standard hygiene precautions with sufficient surveillance or prevalence measurements are performed. Prevalence measurements will provide insight into local HR-GNR epidemiology and possible ongoing transmission within hospitals [27]. Appropriate surveillance could be performed by reviewing (1) clinical HR-GNR isolates, (2) patient admission data and (3) genotyping of HR-GNR isolates when transmission is suspected as performed by Mellmann et al. [30].

Comparing our overall cumulative HR-GNR incidence rate with the study of Willemsen et al. showed a lower cumulative incidence rate per 10,000 admissions (28 vs. 39) [18]. An explanation for this difference could be the large variation between hospitals, hospital types and patient populations that were included in both studies. Comparing the incidence density per 100,000 patient-days between both studies showed a higher incidence density in our study (77 vs. 55) [18]. The mean length of stay in our study was 3.6 days compared to 6.6 days, resulting in a lower denominator of patient-days. This decreasing trend of mean length of stay within Dutch hospitals was also noticed in a Dutch report published in 2013 [34].

The present study has several limitations. First, the sample size (35 index and 69 contact patients) was relatively small which is reflected by the large confidence interval of the calculated transmission rate. Future studies are necessary to confirm our results. Second, for VRE (Vancomycin-resistant *Enterococcus)* it is known that the inoculum size is related to the detection probability with culturing [35]. For HR-GNR detection it is largely unknown how much time between colonization and sampling (using culturing) is sufficient. This may have resulted in a possible underestimation of the nosocomial transmission rate in our study, as some patients could have been marked as false negative. However, the median time between start of contact and sampling in our study was 3 days (median contact time plus time between end of contact and sampling), and we therefore do not think that this has markedly influenced our results. Future studies must incorporate repeated culturing after the end of contact in order to determine the optimal culturing strategy. Third, our results cannot be solely attributed to the transmission capacity of HR-GNR type or micro-organism alone. In a setting, with other prevalence rates or infection control policies other transmission rates could be found. Fourth, we have possibly missed cases of transmission, since only admitted index and contact patients were included in our study.

## Conclusion

In conclusion, our study provides evidence that the nosocomial transmission rate from unexpected HR-GNR positive patients towards short term contacts patients in a local setting within hospitals with standard hygiene precautions is low. In a local setting routine contact tracing among unexpected HR-GNR positive patients may be replaced by appropriate surveillance. As we mainly isolated ESBL *E. coli* and Q&A *E. coli*, our results cannot be extrapolated to other HR-GNR types such as CPE or other micro-organisms such as *K. pneumoniae*.

## Abbreviations

AFLP: Amplification Fragment Length Polymorphism
cgMLST: Core genome multi locus sequence typing
CPE: Carbapenemase Producing Enterobacteriaceae
ESBL: Extended Spectrum Beta Lactamase
HIS: Hospital Information System
HR-GNR: Highly Resistant Gram Negative Rod
ICU: Intensive Care Unit
MDRO: Multi-Drug Resistant Organism
MRSA: Methicillin-resistant *Staphylococcus aureus*
Q&A: Resistant to Fluoroquinolones and Aminoglycosides
RPHLK: Regional Public Health Laboratory Kennemerland
S&D: Search and Destroy
SOP: Standard Operating Procedures
VRE: Vancomycin-resistant *Enterococcus*
wgMLST: Whole genome multi locus sequence typing
WGS: Whole Genome Sequencing
